# Etymologia: *Aspergillus*

**DOI:** 10.3201/eid1203.ET1203

**Published:** 2006-03

**Keywords:** Etymology, Etomoloiga, aspergillus, fungi

## [as´´pər-jil´əs]

**Figure 1:**
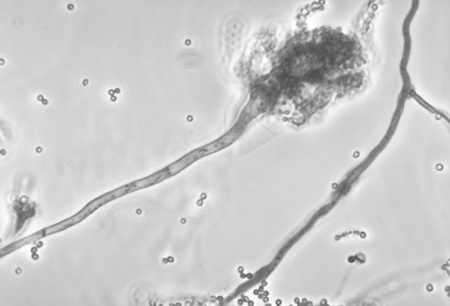
Conidiophore of Aspergillus fumigatus. Image courtesy of Libero Ajello, Centers for Disease Control and Prevention.

**Figure 2:**
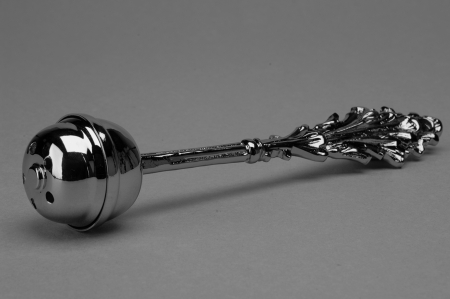
An aspergillum (from the Latin aspergere, "to scatter"), a device used for sprinkling holy water during a liturgical service. Photograph courtesy of Davide Borgonovo.

**Sources:** Dorland's illustrated medical dictionary. 30th ed. Philadelphia: Saunders; 2003 and the Illinois Mycological Association, available from http://www.ilmyco.gen.chicago.il.us/

Genus of filamentous, ubiquitous fungi, commonly isolated from soil, plant debris, and indoor air. *Aspergillus* was first described in 1729 by Pier Antonio Micheli, an Italian priest and biologist who was the first person to attempt the scientific study of fungi. Micheli opposed the idea of "spontaneous generation" by showing that fungal spores grown on a medium would produce the same kind of fungus. The shape of *Aspergillus* (Figure 1) reminded him of an aspergillum (from the Latin *aspergere*, "to scatter"), a device used for sprinkling holy water during a liturgical service (Figure 2).

